# Changes in lysophospholipids and liver status after weight loss: the RESMENA study

**DOI:** 10.1186/s12986-018-0288-5

**Published:** 2018-07-17

**Authors:** Irene Cantero, Itziar Abete, Josep Maria del Bas, Antoni Caimari, Lluís Arola, M. Angeles Zulet, J. Alfredo Martinez

**Affiliations:** 1 Department of Nutrition, Food Science and Physiology, Faculty of Pharmacy and Nutrition, University of Navarra, Irunlarrea 1, 31008 Pamplona, Spain; 2 Centre for Nutrition Research, Faculty of Pharmacy and Nutrition, University of Navarra, Pamplona, Spain; 3 CIBERobn, Physiopathology of Obesity and Nutrition, Instituto de Salud Carlos III, Madrid, Spain; 4 Navarra Institute for Health Research (IdiSNA), Pamplona, Spain; 5 Technological Unit of Nutrition and Health, EURECAT-Technological Center of Catalonia, Reus, Spain; 6 IMDEA Food, Madrid, Spain

**Keywords:** Lysophospholipids, Obesity, Metabolic syndrome, Liver, Hypocaloric diet

## Abstract

**Background:**

Obesity and comorbidities such as non-alcoholic fatty liver disease (NAFLD) are major public health burdens. Alterations in lipid metabolism are involved in hepatic diseases. The objective of this study was to assess the influence of weight loss on lysophospholipid (LP) metabolism and liver status in obese subjects as well as to provide new evidence regarding the interaction of LP metabolism as a key factor in the onset and management of obesity-related diseases such as liver damage.

**Methods:**

Thirty-three subjects from the RESMENA (Reduction of Metabolic Syndrome in Navarra, NCT01087086) study were selected based on their Fatty Liver Index (FLI). Plasma lipid species (lysophosphatidilcholine: LPC, lysophosphatidilethanolamines: LPE and lysophosphatidylinositols: LPI specifically) were determined by LC-MS, while waist circumference (WC) and other non-invasive liver markers such as, FLI and BAAT scores as well as dietary records, anthropometrical measurements, body composition by DXA and other metabolic determinants were analyzed before and after a six-month hypocaloric nutritional intervention.

**Results:**

Computed Z-scores of total LP (LPC, LPE, and LPI) were significantly decreased after 6-months of following a hypocaloric diet. Specifically, LPC14:0, LPC15:0, LPC16:1, LPC18:4, LPC20:4, showed clear relationships with weight loss. Changes in FLI score, WC and BAAT score revealed associations with general changes in LPC score. Interestingly the BAAT score was statistically associated with the LPC score after adjustment for weight loss.

**Conclusion:**

The lipidomic LPC profile analysis revealed a generalized decrease in circulating lysophospholipids after weight loss. The involvement of particular LP in liver metabolism and obesity merit further attention, as some of these specific non-invasive liver markers were reduced independently of weight loss.

**Trial registration:**

NCT01087086. Registered 15 March 2010, retrospectively registry.

**Electronic supplementary material:**

The online version of this article (10.1186/s12986-018-0288-5) contains supplementary material, which is available to authorized users.

## Background

The prevalence of obesity is rising steadily not only in most developed countries but also in transition countries being now estimated that about 1.12 billion adults will be obese by 2030 [1]. Consequently, the prevalence of obesity-associated metabolic diseases such as type 2 diabetes, dyslipidemia, hypertension or hepatic diseases, and accompanying clinical manifestations, will also increase [2, 3]. Current recommendations for obesity management focus on energy-restricted diets with reduced consumption of high energy-dense foods (mainly fat and sugars), as well as higher intakes of fiber and protein to produce satiety, which may be complemented by behavioral or physical activity programs to induce additional weight loss [4]. Also, bariatric surgery and new pharmacological agents may represent appropriate medical strategies for inducing weight loss, but further research is warranted to combat the obesity epidemic.

Fatty liver is generally considered a “benign disease” related to excessive adiposity with low rates of progression to fibrosis, cirrhosis and hepatocellular carcinoma (HCC) [5]. However, given the increasing prevalence of obesity, the incidence of fatty liver-related cirrhosis is gradually on the rise [6]. Healthy dietary pattern strategies leading to weight lowering and maintenance are being considered in the treatment of obesity and associated comorbidities, including non-alcoholic fatty liver disease (NAFLD) and other liver abnormalities [7].

Lipidomics is an emerging technological “omics” discipline focused on the measurement of the relative concentrations of endogenous and exogenous molecules in biofluids, which characterizes changes in metabolism [8] to further understand the metabolic state and dynamic fluxes of biological systems [9]. Several health problems are clearly related to alterations in plasma lipid profiles, including increased fasting triglycerides, low density lipoprotein (LDL) cholesterol, high density lipoprotein (HDL) cholesterol, and elevated blood glucose and insulin levels [10]. Currently, there is no consensus regarding alterations of the plasma lipidome following weight loss in obese subjects. In fact, the mechanisms responsible for the changes observed in the plasma lipidome are unclear and data are limited. Indeed, lipidomics involves establishing relationships between phenotype and metabolism, which are key aspects of biological function [8, 11, 12]. Lysophospholipids (LP) are known to act as signaling molecules and have been linked in different studies to disorders such as fatty liver, steatohepatitis, diabetes, obesity and even cancer. Measurements of these lipids may contribute to understand liver disease and management [13, 14]. However, data regarding alterations of the plasma lipidome following weight loss in individuals with obesity and related disorders remain inconsistent [15].

In this study, we aimed to assess the effects of weight loss on LP metabolism and hepatic status, and to provide new evidence to understand the role of LP metabolism as a key factor in the onset, progression and management of obesity-related diseases such as those related to liver damage. Therefore, we evaluated changes in circulating LP under energy restriction conditions in obese subjects with different degrees of liver status.

## Methods

### Participants and study design

The study plan was approved by the Ethics Committee of the University of Navarra (065/2009). Subjects volunteered to participate in the study and gave their written informed consent following standardized procedures (Additional file 1: Figure S1). The intervention was performed in accordance with criteria and guidelines of the Declaration of Helsinki, and the trial was registered (https://clinicaltrials.gov/ct2/show/NCT01087086; NCT01087086). Details of the study design, participants, and diets were previously reported [16]. A subsample of the Reduction of Metabolic Syndrome (RESMENA) study including 33 Caucasian adults with a body mass index (BMI) greater than 27 kg/m^2^ and at least two features of metabolic syndrome (MetS) as defined by the International Diabetes Federation criteria (IDF) were analyzed [17]. Briefly, the participants were selected based on their FLI index, a noninvasive marker of liver damage [18]. Those participants who fulfilled the requirements for the calculation of FLI were selected and 3 different FLI profiles were calculated to ensure sample representativity. Firstly, those who had a high FLI (≥60) and continued to have higher (≥60) values at the end of the intervention (*n* = 11); secondly, those participants who had a high FLI (≥60), at baseline but whose their FLI values hardly improved (*n* = 12), and thirdly, those subjects who had low values of FLI (< 60) at baseline and also at the end of the intervention (*n* = 10). After a 2-month nutritional-learning intervention period, during which a nutritional assessment was performed on the participants every 15 d, a 4-month self-control period began.

### Nutritional strategy

Briefly, the experimental nutritional intervention consisted of two energy-restricted diets (− 30% of the participant’s requirements) as previously described [16]. The AHA diet was based on the AHA guidelines [19], including three to five meals per day, a designed macronutrient distribution of 55% total caloric value (TCV) from carbohydrate (CHO), 15% TCV from proteins, and 30% TCV from lipids, a healthy fatty acid (FA) profile and a cholesterol content of less than 300 mg/day. The RESMENA diet was designed with a higher meal frequency, consisting of seven meals/day, and a macronutrient distribution of 40% TCV from CHO (whole grains were required), 30% from proteins (mainly vegetable protein), and 30% from lipids (omega-3 and extra virgin olive oil intake required) as reported elsewhere [16].

### Analysis of plasma phospholipids by LC-MS

Fasting plasma LP were analysed as previously detailed [14]. Briefly, samples were extracted with methanol: water using LPC13:0 as an internal standard. LP were separated by reverse phase liquid chromatography using an Agilent 1290 Infinity HPLC system equipped with a ZORBAX C18 SB-Aq 2.1-mm 3 50-mm, 1.8-mm particle analytic column (Agilent Technologies). An Agilent ZORBAX C-8 2.1-mm 3 30- mm, 3.5-mm particle guard column was placed in series in front of the analytic column. An Agilent 1290 Infinity HPLC system with a binary pump and degasser, thermostated well plate autosampler, and column compartment were used. The autosampler temperature was 4 °C, the injection volume was 2 μL, the column temperature was 60 °C, and the flow rate was 0.6 mL/m. The repeatability (RSD) or coefficients of variation were as follows: LPC14:0 (5.8%); LPC15:0 (6.2%); LPC16:0 (4.7%); LPC16:1 (4.6%); LPC17:0 (4.2%); LPC17:1 (5.1%); LPC18:0 (3.4%); LPC18:1 (4.5%); LPC18:2 (4.6); LPC18:3 (8.3%); LPC18:4 (3.7%); LPC20:0 (3.4%); LPC20:1 (3.6%); LPC20:2 (5.5%); LPC20:3 (6.7%); LPC20:4 (8.1%); LPC22:4 (3,0%); LPC22:5 (5.6%); LPC22:6 (7.9%). LP species were identified by mass spectrometry. An Agilent 6550 Accurate-Mass Quadrupole-Time of Flight (Q-TOF) mass spectrometer (MS) (Agilent Technologies, Santa Clara, CA, USA) was operated in ESI+ and ESI- modes. Calibration curves were constructed using 1 to 1500 μg/L LPC16:0, LPC18:0, LPC20:0, LPE18:1, and LPI18:1 as standards. An LPC16:0 calibration curve was used to quantify LPC14:0, LPC16:0 and LPC16:1; an LPC18:0 calibration curve was used to quantify LPC18:0, LPC18:1, LPC18:2, LPC18:3 and LPC18:4; and LPC20:0 was used to quantify LPC20:0, LPC20:1, LPC20:2, LPC20:3, LPC20:4, and LPC22:5. All LPE and LPI species were quantified using the calibration curves of LPE18:1 and LPI18:1, respectively. The limit of detection was set at 0.04 μM.

### Anthropometric and biochemical measurements

Anthropometric measurements, such as body weight and waist circumference (WC), were determined in fasting conditions following previously described standardized procedures [20]. Visceral Adipose Tissue (VAT) and body composition were assessed by dual-energy x-ray absorptiometry (Lunar Prodigy, software version 6.0, Madison, WI) at baseline and after 6 months with the subjects in their underwear in accordance with validated protocols [21]. BMI was calculated as body weight divided by squared height (kg/m^2^). Glucose, total cholesterol (TC), triglycerides (TG), Alanine aminotransferase (ALT), Aspartate aminotransferase (AST) and Gamma-glutamyl transferase (GGT) were measured with an autoanalyzer (Pentra C-200; HORIBA ABX, Madrid, Spain) using specific kits (provided by Mercodia, Upssala, Sweden). Plasma concentrations of CK18-fragments (M30 and M65) were assessed by an ELISA assay with the same autoanalyzer system (Triturus, Grifols SA, Barcelona, Spain) in accordance with the manufacturer’s instructions.

A number of non-invasive markers and indices of NAFLD were used to assess liver status. The Fatty Liver Index (FLI) is an algorithm derived from serum TG, BMI, WC and GGT values [22–25], which has been validated in a large group of subjects at high risk for developing fatty liver disease. The FLI [18], a surrogate marker of NAFLD prognosis, was estimated as follows: FLI  =  (e0.953  ×  loge [TG]  +  0.139  ×  BMI  +  0.718  ×  loge [GGT]  +  0.053  ×  WC  −  15.745)/(1  +  e0.953  ×  loge [TG]  +  0.139  ×  BMI  +  0.718  ×  loge [GGT]  +  0.053  ×  WC  −  15.745)  ×  100. An index of < 30 points indicates the absence of fatty liver and an index ≥60 is a marker of fatty liver. The NAFLD liver fat score (NAFLD_LFS) [26] was calculated by an equation combining the following variables: the presence of MetS (as defined by IDF criteria), the presence of Type 2 diabetes, fasting serum insulin, AST, and the aspartate-alanine aminotransferase ratio (AST/ALT ratio) as follows: − 1.675 + 0.037 × age (years) + 0.094 × BMI (kg/m2) + 1.13 × IFG/diabetes (yes = 1, no = 0) + 0.99 × AST/ALT ratio – 0.013 × platelet (× 109/l) – 0.66 × albumin (g/dl). This score predicts the presence of steatosis, defined as a liver fat content ≥5.56%, as assessed by 1H-Magnetic Resonance Spectroscopy (1H-MRS) with good accuracy (area under the receiver operating characteristic curve [AUROC]: 0.86). The Hepatic Steatosis Index (HSI) was estimated using a formula including the ALT/AST ratio, the presence of diabetes and gender: (HSI) = 8 x (ALT/AST ratio) + BMI (+ 2, if female; + 2, if diabetes mellitus). HSI values < 30 and > 36 signify the presence and absence of fatty liver, respectively, with a diagnostic accuracy of 93.1% [27]. The Visceral Adipose Index (VAI) is another marker [28], which was calculated with different formulas for men and women (Males = [WC/39.68 + (1.88 × BMI)] × (TG/1.03) × (1.31/HDL) and Females = [WC/36.58 + (1.89 × BMI)] × (TG/0.81) × (1.52/HDL). Based on the available data, three predictors of hepatic steatosis were also computed. Firstly, we calculated the BAAT score (BMI, ALT AGE and TG) [29], which includes: BMI (≥28 = 1, < 28 = 0), age (≥50 years = 1, < 50 = 0), ALT [≥2 UNL (males, ALT ≥60 IU/L; females, ALT ≥40 IU/L) = 1, < 2 UNL = 0) and serum triglycerides [1.7 mmol/L (=150 mg/dL) = 1, < 1.7 = 0]. The second predictor is the BARD score (BMI, AST/ALT ratio and Diabetes) as described elsewhere [30], but which includes a BMI (≥28–1 point), the AST/ALT ratio (≥0.8–2 points) and the presence of diabetes (− 1 point). The possible score ranges from 0 to 4 points. Lastly, we used the TyG-index (TG/glucose index) which has been described and validated as a predictor of hepatic steatosis with a threshold of ≥8.5, and effectively identifies the presence of NAFLD, with an AUC of 0.782 [31].

### Statistical analysis

Statistical tests were performed using STATA version 12.0 (Stata Corp). The sample size of the RESMENA study was calculated as previously reported [16]. Normality distributions of the variables measured were determined with the Shapiro-Wilk test. Most of the lysophospholipid data had a normal distribution with the exception of LPC14:0, LPC20:2, LPE18:2, LPE20:2 and LPE20:5, for the evaluation of which non-parametric tests were used. Continuous variables were compared between groups by the Student’s t-test or the Mann–Whitney U test for parametric or non-parametric distributions, respectively. Bonferroni adjustment (Post Hoc test) was applied, which implied a correction for multiple comparison, whose corrected *p*-values are given in the table as appropriate. We penalized the uncorrected *p*-value using the formula p’ = 1-(1-p)^c, where p’ is the penalized *p*-value, p is the uncorrected *p*-value and c is the number of comparisons. Corrected *p*-values are presented throughout the paper [32]. Categorical variables were compared by the chi-squared test. Tables 1 and 2 were categorized by the median value (< P50 vs ≥ P50) concerning changes in WC and weight loss. Standardized data on LP concentrations were obtained to calculate z-scores. Standardization is a simple procedure: [(Value – mean)/SD], where the objective is to express a variable in the number of standard deviations separating a value from the total average.

**Table 1:** Characteristics of the participants categorized by changes of P_50_ of WC and weight loss of the participants

	All subjects			Δ Waist circumference (cm)		Δ Weight (kg)	
*n* = 33	Baseline	6-months	P	< P50	≥ P50	< P50	≥ P50
Δ Anthropometry							
Weight (kg)	100.1 (9)	91.2 (19)	< 0.001	−6.1 (2)	− 11.2 (4)	−5.0 (2)	− 12.3 (33)
BMI (kg/m^2^)	34.8 (4)	31.7 (5)	< 0.001	*− 2.2 (1)*	−3.9 (1)*	− 1.8 (0.9)*	−4.4 (1)*
WC (cm)	110 (13)	102 (14)	< 0.001	*−4.8 (2)*	*− 12.3 (3)*	−5.6 (3.5)*	− 11.5 (4)*
% TFM	37 (10)	31 (9)	< 0.001	*−4.5 (3)*	−8.6 (3)*	−4.0 (2)	−9.1 (3)
VAT (kg)	3.1 (1.7)	2.2 (1)	< 0.001	− 0.8 (0.9)	− 0.9 (1)	− 0.8 (0.9)	− 0.9 (1)
Δ General biochemistry							
Glucose (mg/dl)	121.5 (37)	117.0 (32)	0.118	−1.2 (13)	−7.6 (18)	−2.4 (9)	−6.4 (20)
Homa-IR	4.4 (3)	2.5 (3)	< 0.001	−1.7 (2)	−1.8 (15)	− 1.7 (2)	−1.8 (1)
TC (mg/dl)	379 (144)	272 (106)	0.001	−55.3 (154)	− 155.1 (172)	−70.0 (24)	− 141.3 (199)
HDL (mg/dl)	46.5 (10)	49.0 (10)	0.069	2.8 (7)	2.0 (7)	4.7 (6)	0.07 (8)
LDL (mg/dl)	136.0 (44)	167.3 (38)	0.001	46.6 (37)	14.0 (57)	43.8 (35)	17.7 (60)
TG (mg/dl)	198.6 (120)	160.4 (116)	0.003	−16.4 (74)	−61.2 (59)	−28.5 (75)	−48.3 (64)
TyG -index	9.1 (0.7)	8.9 (0.7)	< 0.001	−0.1 (0.3)	−0.3 (0.3)	−0.2 (0.3)	− 0.3 (0.3)
SBP (mmHg)	153.3 (21)	140.7 (15)	0.003	−12.9 (20)	−12.1 (24)	−7.1 (21)	− 18.3 (23)
DBP(mmHg)	84.4 (9)	79.4 (10)	0.022	−5.7 (9)	−4.0 (14)	−2.6 (10)	−7.2 (12)
Δ Hepatic status							
ALT (U/L)	34.9 (21)	23.1 (8)	0.001	−9.3 (15)	−14.2 (23)	− 13.0 (15)	− 10.3 (24)
AST (U/L)	26.0 (11)	21.3 (5)	0.027	−2.1 (9)	−7.5 (13)	−5.3 (10)	−4.1 (13)
GGT (U/L)	45.6 (32)	31.6 (19)	< 0.001	−13.1 (18)	−14.8 (17)	− 15.8 (19)	−11.9 (16)
FLI	84.7 (18)	64.9 (27)	< 0.001	*−10.1 (11)*	−30.0 (16)*	*− 10.6 (10)*	−29.4 (17)*
M30 (U/L)	199.9 (14)	123.9 (48)	0.002	−57.6 (110)	−45.4 (145)	−82.5 (152)	−69.0 (106)
M65 (U/L)	325.7 (206)	213.7 (72)	0.002	−79.6 (149)	− 146.2 (241)	−116.5 (215)	− 107.0 (187)
HSI	47.7 (6)	42.6 (6)	< 0.001	−4.2 (3)	−5.9 (3)	− 4.1 (36)	−6.0 (3)
VAI	3.2 (2)	2.4 (2)	0.008	−0.5 (1)	−1.2 (1)	−0.7 (1)	−0.9 (1)
NAFLD_LFS	1.8 (2)	0.2 (2)	< 0.001	−1.2 (1)	−1.9 (1)	− 1.5 (1)	−1.6 (2)
BARD score	2.7 (1)	2.9 (1)	0.587	0.5 (1)	−0.3 (1)	0.4 (1)	−0.1 (1)
BAAT score	2.1 (0.7)	1.7 (0.6)	< 0.001	−0.2 (0.4)	−0.6 (0.6)	− 0.3 (0.4)	−0.5 (0.6)
Δ Inflammatory status							
hsCRP (mg/l)	4.6 (6)	2.6 (3)	0.035	−2.2 (6)	−1.8 (3)	−1.2 (2)	−2.9 (7)

(mean ± SD). Pared and independent t tests were carried out. *p < 0.05, comparison within each group (<WC/weight P50 vs ≥ WC/weight P50). *P*-values were adjusted using an ancova test adjusting for sex and gender

*BMI* Body Mass Index, *WC* Waist Circumference, *TFM* Total Fat Mass, *VAT* Visceral Adipose Tissue, *SBP* Systolic Blood Pressure, *HOMA-IR* Insulin Resistance HOMA index, *DBP* Diastolic Blood Pressure, *TC* Total Cholesterol, *HDL* High Density Lipoprotein, *LDL* Low Density Lipoprotein, *TyG* triglycerides/glucose ratio, *TG* Triglycerides, *ALT* Alanine Transaminase, *AST* Aspartate Transaminase, *GGT* Gamma-glutamyl transferase, *HSI* hepatic steatosis index, *VAI* Visceral adipose index, *NAFLD_LFS* Fatty liver disease, BARD score; *BAAT* score and *hsCRP* his sensitivy C-reactive protein

**Table 2:** Changes in lysophospholipids concentration (μM) categorized by changes in waist circumference and Weight loss

	All subjects (*n* = 33)			Δ Waist circumference (cm)		Δ Weight (kg)	
Δ LP	Baseline	6 months	*p*-value	< P50	≥ P50	< P50	≥ P50
LPC 14:0	1.7 (0.1)	1.2 (0.1)	0.008	0.5 (1)	1.1 (1)	0.8 (1)	0.8 (1)
LPC 15:0	0.8 (0.05)	0.7 (0.04)	0.024	*0.3 (0.5)*	0.7 (0.4)*	0.4 (0.6)	0.6 (0.4)
LPC 16:0	147 (6)	136 (8)	0.231	*83.2 (101)*	158.0 (75)**	108.1 (67)	137.9 (112)
LPC 16:1	3.7 (0.2)	3 (0.19)	0.009	*1.1 (2.4)*	8.3 (1)*	2.1 (2)	2.4 (2)
LPC 17:0	3 (0.2)	2.6 (0.2)	0.105	*1.1 (2)*	3.1 (1)*	1.8 (2)	2.5 (2)
LPC 17:1	0.6 (0)	0.5 (0)	0.095	0.3 (0.4)	0.5 (0.2)	0.4 (0.4)	0.4 (0.3)
LPC 18:0	78.6 (4.8)	65.5 (5)	0.060	*29.0 (66)*	74.3 (51)*	37.2 (57)	66.6 (65)
LPC 18:1	55.8 (4.1)	49.8 (3.3)	0.189	*24.9 (32)*	61.6 (43)*	49.5 (34)	51.0 (48)
LPC 18:2	56.8 (3.2)	57 (4)	0.856	47.7 (48)	68.1 (43)	49.5 (34)	66.6 (54
LPC 18:3	81.4 (3.7)	77.4 (5.3)	0.503	*50.0 (63)*	92.6 (50)*	66.2 (38)	79.3 (72)
LPC 18:4	1.1 (0.06)	0.89 (0.05)	0.006	*0.3 (0.6)*	0.9 (0.4)*	0.6 (0.6)	0.7 (0.6)
LPC 20:0	0.31 (0.02)	0.29 (0.02)	0.547	0.2 (0.3)	0.2 (0.2)	0.3 (0.2)	0.2 (0.2)
LPC 20:1	0.84 (0.06)	0.74 (0.04)	0.124	*0.4 (0.5)*	0.8 (0.4)*	0.5 (0.4)	0.7 (0.5)
LPC 20:2	0.83 (0.06)	0.7 (0.04)	0.090	*0.3 (0.6)*	0.8 (0.3)*	0.4 (0.6)	0.6 (0.5)
LPC 20:3	9.94 (0.6)	8.77 (0.56)	0.214	6.0 (9)	9.0 (6)	7.2 (7)	7.8 (8)
LPC 20:4	36.5 (2)	31 (1.7)	0.026	*16.2 (16)*	34.5 (18)*	21.2 (17)	30.2 (21)
LPC 22:4	0.43 (0.02)	0.39 (0.02)	0.222	0.2 (0.3)	0.4 (0.2)	0.3 (0.3)	0.3 0.2)
LPC 22:5	1 (0.07)	0.98 (0.05)	0.256	0.7 (0.8)	0.9 (0.7)	0.8 (0.7)	0.8 (0.7)
LPC 22:6	7.8 (0.5)	6.6 (0.5)	0.093	0.7 (0.8)	0.9 (0.7)	0.9 (0.8)	0.8 (0.9)
LPE 18:0	25.7 (1.7)	23 (1.5)	0.230	15.1 (20)	24.9 (17)	20.6 (19)	20.0 (20)
LPE 18:1	3.8 (0.36)	3.57 (0.3)	0.627	3.1 (4)	3.5 (4)	3.5 (4)	3.1 (5)
LPE 18:2	4.1 (0.3)	3.9 (0.4)	0.803	4.2 (6)	3.4 (4)	4.0 (5)	3.6 (5.0)
LPE 20:0	6.7 (0.58)	5.7 (0.49)	0.202	3.3 (6)	6.1 (5)	4.9 (5)	4.8 (6)
LPE 20:2	5.2 (0.39)	5.1 (0.4)	0.911	4.4 (5.5)	5.7 (5)	4.7 (5)	5.4 (5)
LPE 20:3	0.43 (0.01)	0.42 (0.01)	0.872	0.4 (0.2)	3.0 (2)	0.4 (0.2)	0.4 (0.2)
LPE 20:4	3.37 (0.16)	3.15 (0.21)	0.427	2.8 (3.1)	0.2 (0.8)	3.1 (2)	2.7 (2)
LPE 20:5	0.75 (0.06)	0.77 (0.16)	0.917	1.3 (2.4)	0.5 (0.2)	1.1 (2)	0.4 (0.9)
LPE 22:4	0.46 (0.01)	0.48 (0.02)	0.332	0.4 (0.2)	0.5 (0.2)	0.4 (0.2)	0.5 (0.3)
LPE 22:5	0.27 (0.0)	0.27 (0.0)	0.931	0.2 (0.1)	0.2 (0.09)	0.2 (0.09)	0.2 (0.1)
LPE 22:6	1.9 (0.13)	1.66 (0.1)	0.054	1.05 (1)	1.6 (0.9)	1.5 (1)	1.1 (1)
LPI 18:0	2.5 (0.2)	2.2 (0.23)	0.367	1.2 (3)	2.5 (2)	1.7 (3)	2.1 (2)
LPI 18:1	0.61 (0.06)	0.5 (0.03)	0.105	0.3 (0.5)	0.4 (0.4)	0.4 (0.4)	0.3 (0.5)
Scores of lysophospholipids							
∑LPC-s	*2.5 (14)*	−5.4 (13)*	0.027	*0.1 (0.5)*	−0.8 (1)*	−0.5 (0.1)	−0.1 (0.8)
∑LPE-s	0.5 (7)	−0.8 (8)	0.495	−0.2 (1)	−0.2 (1)	−0.1 (1)	−0.03 (1)
∑LPI-s	0.2 (1)	−0.3 (1)	0.178	−0.65 (1)	−0.03 (1)	− 0.4 (1)	−0.2 (1)
∑LPstotal	*2.6 (19)*	−9.2 (20)*	0.024	*−0.07 (0.5)*	−0.6 (0.8)*	− 0.2 (0.6)	−0.5 (0.8)

Paired test was carried out. Mean (SD). * *P*-values considered < 0.05 and **P-values were *p* < 0.001. *P*-values were adjusted by Ancova test was performed adjusting by LP value at baseline, sex and gender. ∑LPCs is lysophosphatidylcholine score, ∑LPEs is lysophosphatidylethanolamine score; ∑LPIs is lysophosphatidylinositols score; ∑LPstotal is lysophospholipis total score

A linear regression model was set up to assess the influence of independent variables such as lysophosphatydilcholine (LPC) with the FLI, WC and the BAAT score (adjusted by baseline LP values, age and gender). All *p*-values presented are two-tailed, and differences were considered statistically significant at *p* < 0.05, while the Bonferroni correction was applied as appropriate.

## Results

After six months, both dietary treatments were effective at improving most of the variables related to body composition, general biochemistry and hepatic status (Table 1). The average sample age was 50.5 years, and the sample was made up of 59.3% men. Participants showed metabolic syndrome in 80% of the cases according to IDF criteria [17] and 12% were smokers. In addition, subjects with better improvements in WC and weight loss achieved greater changes in anthropometric, general biochemistry and hepatic status measurements. At baseline 1 patient had an FLi < 30, 3 had FLI ≥30 and < 60 and 29 patients had FLI ≥ 60 while at the end of the intervention, the value distribution of this hepatic marker respectively were 4, 9, 20. It is important to note that 8 participants did not achieve a 5% weight loss, 12 subjects achieved a 5% weight loss and 13 achieved a 10% weight loss.

**Fig. 1:**
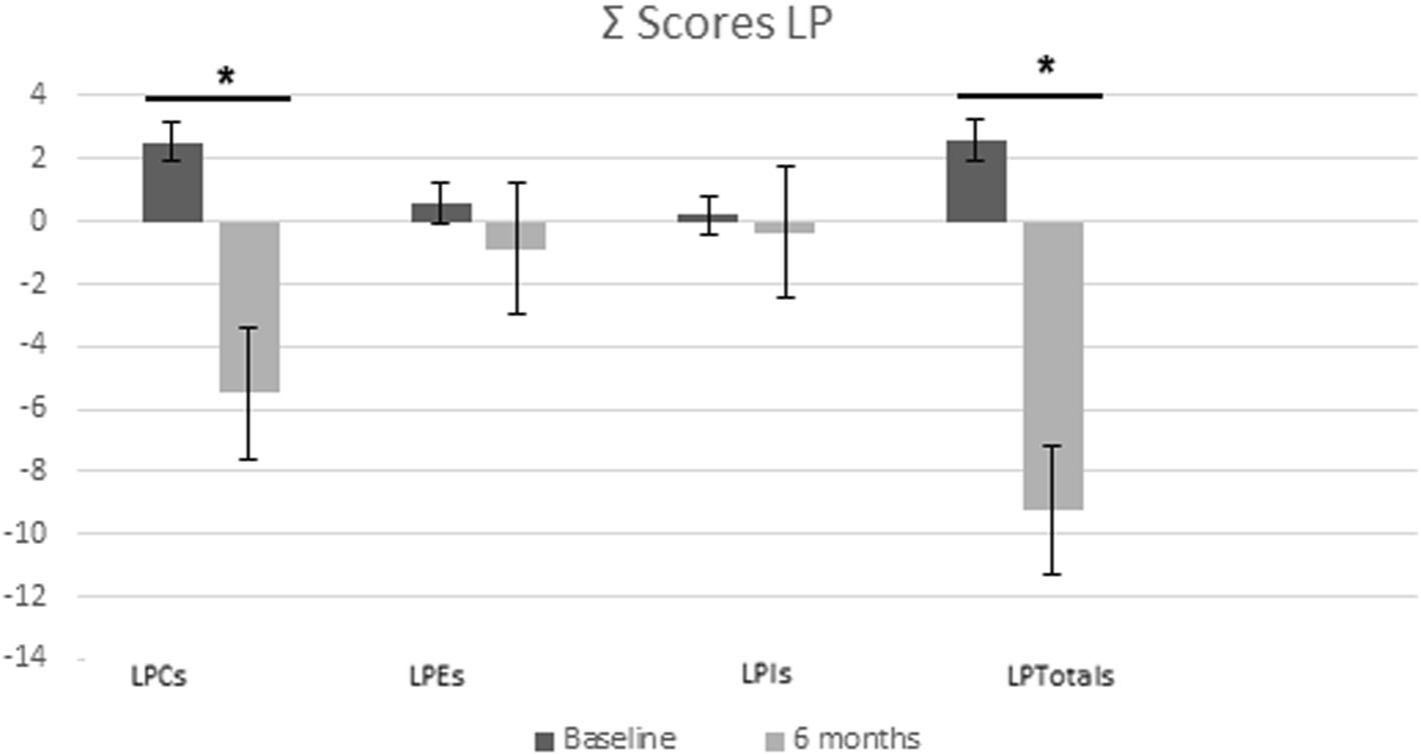
Changes in LP scores at baseline and after 6 months

Circulating concentrations of each LP were determined via lipidomic analysis (Table 2). In the intervention groups, most circulating LP concentrations decreased by the end of the intervention and specifically in five LPC (LPC14:0, LPC15:0, LPC16:1, LPC18:4, LPC20:4) the decreases were statistically significant. Thus, we decided to analyze them further (Table 3). A number of circulating LP concentrations belonging to the choline group decreased when categorizing subjects by changes in waist circumference, patients with more significant improvements in WC, and those who had larger reductions in LPC. Although patients with greater weight loss changes showed improvements in their LPC profiles, these findings were not statistically significant. Regarding summarized z-scores which included the lisophospatidylcholine score (LPC score), lysophosphatidylethanolamines score (LPE score), lysophosphatidylinositol score (LPI score) and lysophopolipid total score (LPTotals), only the LPC score and LPTotals showed an overall significant reduction after 6 months of nutritional intervention (Fig. 1).

**Table 3:** Linear regression between changes in FLI, BAAT and WC with specific LPC concerning both intervention treatments taking into account sex, age and dietary group

(*n* = 33)	β	p	P _model_	R^2^
Δ FLI				
Δ LPC14:0	5.58	0.078	0.066	0.167
Δ LPC15:0	7.19	0.308	0.037	0.209
Δ LPC16:1	3.49	0.096	0.028	0.221
Δ LPC18:4	12.27	0.111	0.033	0.210
Δ LPC20:4	0.28	0.753	0.047	0.185
Δ BAAT				
Δ LPC14:0	0.45	0.010	0.016	0.274
Δ LPC15:0	0.50	0.154	0.327	0.029
Δ LPC16:1	0.12	0.209	0.509	0.017
Δ LPC18:4	0.47	0.205	0.487	0.013
Δ LPC20:4	0.00	0.902	0.789	0.032
Δ WC				
Δ LPC14:0	−0.00	0.957	0.441	0.004
Δ LPC15:0	4.93	0.162	0.044	0.196
Δ LPC16:1	0.85	0.133	0.332	0.025
Δ LPC18:4	3.13	0.165	0.336	0.024
Δ LPC20:4	0.13	0.031	0.112	0.121

Models were adjusted for sex, age and dietary group. *FLI* Fatty Liver Index; BAAT score, *WC* Waist Circumference, *LPC14:0* Lysophosphatidylcholine 14:0, *LPC15:0* Lysophosphatidylcholine 15:0, *LPC16:1* Lysophosphatidylcholine 16:1, *LPC18:4* Lysophosphatidylcholine 18:4, *LPC20:4* Lysophosphatidylcholine 20:4

**Fig. 2:**
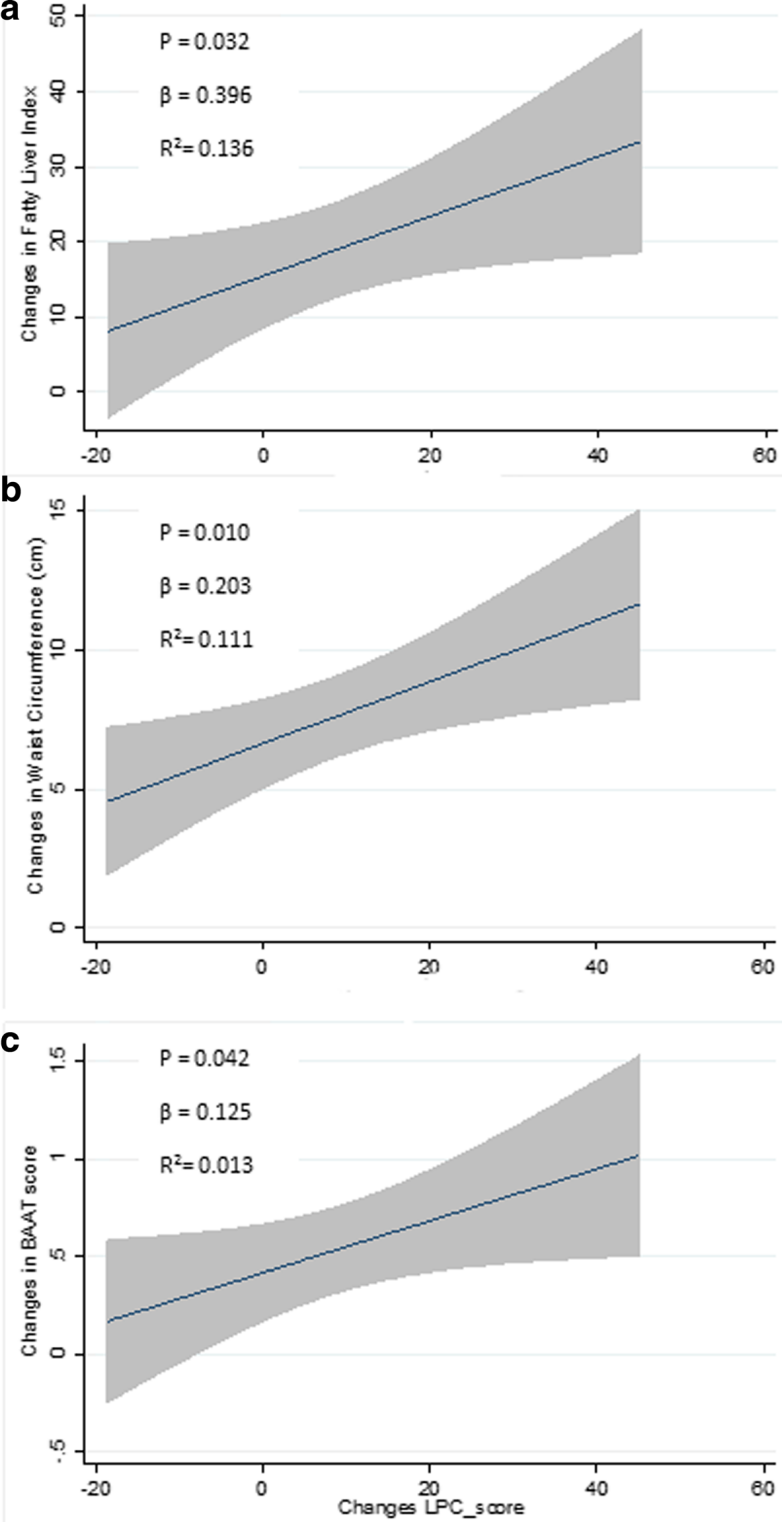
Linear regression plots between changes in LPC score with FLI, BAAT and waist circumference

Linear regression analyses revealed positive and significant associations (Fig. 2) between changes in LPC score and changes in WC (β = 0.203, *p* = 0.010, R^2^ = 0.111) and changes in two non- invasive of liver markers: the fatty liver index (β = 0.396 *p* = 0.032, R^2^ = 0.136) and the BAAT score (β = 0.125, *p* = 0.042, R^2^ = 0.013).

In addition, linear regression analyses (Table 3) showed positive and significant association between changes in FLI and BAAT and WC with changes in specific LPC group (LPC14:0, LPC15:0, LPC16:1, LPC18:4, LPC20:4). Specifically, FLI was associated with LPC16:1 (β = 4.494 *p* = 0.024, R2 = 0.130); LPC18:4 (β = 15.71 *p* = 0.033, R2 = 0.113); the BAAT score with: LPC14:0 (β = 0.331 *p* = 0.010, R2 = 0.182); LPC:15 (β = 0.519 *p* = 0.049, R2 = 0.100); and WC with LPC15:0 (β = 4.720 *p* = 0.011, R2 = 0.174); LPC16:1 (β = 1.032 *p* = 0.045, R2 = 0.097); LPC18:4 (β = 3.771 *p* = 0.048, R2 = 0.124); LPC20:4 (β = 0.130 *p* = 0.030, R2 = 0.118). When we assessed the same analysis after adjusting for the percentage of weight loss (categorized by ≥5% and ≥ 10%), associations between the changes in FLI and changes in two different LPC, LPC16:1 (β = 2.920 *p* = 0.040, R^2^ = 0.575) and LPC18:4 (β = 10.41 *p* = 0.048, R^2^ = 0.570) were found. Also, changes in the BAAT score were related to changes in LPC14:0 (β = 0.285 *p* = 0.024, R^2^ = 0.250) and changes in WC were associated with LPC15:0 (β = 3.321 *p* = 0.032, R^2^ = 0.445). Interestingly, when the data were adjusted by the percentage of weight loss, a higher impact of total LP on FLI (β = 0.09, *p* = 0.027, R^2^ = 0.362) and WC (β = 0.162, *p* = 0.279, R^2^ = 0.5334) was identified with the exception of the BAAT score. Most of the LP analyzed in this study showed a positive significant association with a weight loss of ≥10% (Table 4). The range of R2 values varied from 0.004 to 0.274 (Table 3), when adjusting for sex, age and dietary group and from 0.049 to 0.575 when additionally adjusted by for weight loss (Table 4). The contribution of adjusting variables without lysophospholipids was: 0.161, 0.044, 0.021 for FLI, BAAT and WC respectively (Table 3). Likewise, the R2 values in Table 4, which only take into account adjusted variables (age, sex, dietary treatment and % of weight loss) were: 0.502, 0.024, 0.213 for FLI, BAAT and WC, respectively.

**Table 4:** Linear regression analyses between changes in FLI, BAAT and WC with specific Lysophosphatidylcholine adjusted for the percentage of weight loss in addition to sex, age and dietary group

*n* = 33	β	p	R^2^	P _model_
Δ FLI				
Δ LPC14:0	4.61	0.070	0.545	< 0.001
Weight loss (5%)	13.55	0.016		
Weight loss (10%)	30.8	< 0.001		
Δ LPC15:0	−1.02	0.859	0.510	< 0.001
Weight loss (5%)	13.94	0.018		
Weight loss (10%)	31.51	< 0.001		
Δ LPC16:1	2.92	0.040	0.575	< 0.001
Weight loss (5%)	12.77	0.018		
Weight loss (10%)	29.33	< 0.001		
Δ LPC18:4	10.41	0.048	0.570	< 0.001
Weight loss (5%)	13.27	0.015		
Weight loss (10%)	29.67	< 0.001		
Δ LPC20:4	0.21	0.204	0.532	< 0.001
Weight loss (5%)	13.49	0.018		
Weight loss (10%)	30.60	< 0.001		
Δ BAAT				
Δ LPC14:0	0.28	0.024	0.250	0.014
Weight loss (5%)	0.13	0.563		
Weight loss (10%)	0.47	0.053		
Δ LPC15:0	0.41	0.126	0.115	0.105
Weight loss (5%)	0.11	0.0646		
Weight loss (10%)	0.38	0.144		
Δ LPC16:1	0.00	0.384	0.062	0.183
Weight loss (5%)	0.08	0.740		
Weight loss (10%)	0.41	0.123		
Δ LPC18:4	0.23	0.380	0.069	0.182
Weight loss (5%)	0.09	0.707		
Weight loss (10%)	0.42	0.116		
Δ LPC20:4	0.00	0.630	0.049	0.230
Weight loss (5%)	0.09	0.703		
Weight loss (10%)	0.45	0.098		
Δ WC				
Δ LPC14:0	0.24	0.759	0.276	0.008
Weight loss (5%)	0.33	0.849		
Weight loss (10%)	5.49	0.004		
Δ LPC15:0	3.32	0.032	0.445	< 0.001
Weight loss (5%)	−0.63	0.662		
Weight loss (10%)	4.31	0.006		
Δ LPC16:1	0.72	0.124	0.274	0.007
Weight loss (5%)	0.29	0.864		
Weight loss (10%)	4.43	0.016		
Δ LPC18:4	2.63	0.130	0.272	0.007
Weight loss (5%)	0.41	0.808		
Weight loss (10%)	4.52	0.014		
Δ LPC20:4	0.09	0.069	0.299	0.004
Weight loss (5%)	0.62	0.713		
Weight loss (10%)	4.50	0.011		

Models were adjusted for weight loss, age and sex. *FLI* Fatty Liver Index, *BAAT* BAAT score, *WC* Waist Circumference, *LPC14:0* Lysophosphatidylcholine 14:0, *LPC15:0* Lysophosphatidylcholine 15:0; *LPC16:1* Lysophosphatidylcholine 16:1, *LPC18:4* Lysophosphatidylcholine 18:4, *LPC20:4* Lysophosphatidylcholine 20:4

## Discussion

Obesity and related comorbidities are a major health concern that require new approaches to understanding this metabolic disease, [2] including the design of new dietary strategies for MetS prevention and precision management of causal factors [33]. Because circulating lipids play an important role in obesity and metabolic syndrome manifestations, current research is focused on quantitative plasma lipid profiling in obesity and subsequent weight loss [34]. This study investigated plasma lipid changes following a 6-month dietary intervention with two energy-restricted diets containing a different distribution of macronutrients focusing on anthropometrics, biochemical measurements, and non-invasive markers of liver status in obese individuals with MetS features. Both diets were equally effective in inducing weight loss and most body composition changes with the exception of total fat mass, a finding that is in agreement with similar nutritional intervention studies [35]. Surprisingly, LDL-c increased. This result, however, is consistent with findings from, different systematic reviews that report no clear effects of hypoenergetic diets on LDL-C depletion [36, 37] and with studies including the RESMENA project that state that in some cases LDL-C values may increase despite weight loss [16, 38]. The positive effects on weight loss after 6 months were found concerning the reduction of total visceral fat and adipose tissue, a fat region that has been commonly associated with hepatic steatosis [39]. Current non-invasive evaluations of NAFLD could be useful for the assessment and monitoring of fatty liver conditions [40], with MRI imaging being the best method for highly accurate non-invasive measurement of liver steatosis [41]. In this context, Bedogni et al. developed a simple scoring system called FLI, which involves different markers (TG, GGT, BMI, WC), which may help physicians to intensify lifestyle counseling and researchers to select patients for epidemiologic studies in relation to NAFLD. However, validation of FLI in external populations is needed before it can be employed for these purposes [18]. Several studies [27, 28, 31, 42, 43] have validated the non-invasive markers of liver status that were used in this study (CK18-fragments, HSI, VAI, Triglycerides/glucose index, BARD and BAAT score, and NAFLD_LFS).

Targeted LC-MS lipid profiling used in the present study proved to be a useful approach to identify major differences in plasma lipids after a long-term dietary intervention [14]. Most of the markers identified were lipid species, primarily glycerophospholipids, such as phosphatidylcholines and a LPC score. In this context, lysophospholipids were significantly reduced after weight loss and when *p*-values were adjusted for baseline LP values, some p-values lost significance, but in all cases statistical trends values were obtained. Therefore, LPC14:0, LPC15:0, LPC16:1, LPC18:4 and LPC20:4 showed a significant reduction, possibly due to the effect of weight loss [34]. The literature to date remains inconsistent concerning to the relation between weight loss in obese individuals and circulating LP concentrations. In this study, an association between LPC16:4, LPC18:4 and FLI was found even after adjusting for percentage of weight loss, giving new views about the importance of the role of weight lowering on managing liver disease in well-designed nutritional interventions. Thus, we cannot be sure if the reduction in LP is due only to the weight loss or if any component of the diet could be interacting with it or both factors are at play. In another study of diet-induced weight loss in obese individuals, a reduction of serum LPC and triacyclglycerols, predominantly short chain fatty acids, was observed, while other lipid classes such as sphingolipids and LPC remained unaffected by weight loss [44]. In contrast, our study showed an overall reduction of total LP after weight loss. The total LP value may have reached significance due to the effect of the choline group, as no significant results were found for LPI and LPE individually. In agreement with our results, a human study concerning lipidomic profiling revealed a generalized decrease in circulating LPC species after weight loss in the obese state. Furthermore, the authors identified LPC subjects (LPC16:0, LPC18:0 among others) as potential markers for obesity through profiling of plasma [15].

Another study [15] reported that LPC levels are decreased significantly in obese subjects both before and after weight loss. This outcome was attributed to lower plasma levels of nearly all LPC species, particularly LPC 16∶0, 18∶0, 18∶1 and 18∶2. Also, Barber et al. [34] reported decreased plasma concentrations of total LPC, LPC 15∶0, 18∶0, 18∶1, 18∶2 and 20∶4 in states of obesity and MetS.

Different trends have been observed for LPC species in other studies. One study on acquired obesity in monozygotic twins revealed an association between obesity and increased LPC levels, while ether phospholipids were found to be decreased [45]. Similarly, Graessler et al. compared the plasma lipidome of men with a BMI > 27.5 kg/m^2^ relative to an AHA diet group of men with BMI < 27.5 kg/m^2^ and found a positive association between LPC species and higher BMI levels. However, in this trial statistical significant differences were only detected for LPC 16∶0 [46]. These findings provide insights into the mechanisms that favor the progression of metabolic syndrome and obesity. A limitation of this study is that NAFLD was evaluated using non-invasive markers instead of imaging techniques and / or liver biopsies. However, the design of the current tests are based on some non-invasive markers, which are suitable for use in a research study such as this trial [47]. Regarding sample size, the participants of this investigation were a subsample from the RESMENA study. This study was designed taking into account only two hypocaloric dietary groups without normo-caloric diet working as control group, which could limit the generalizability of our findings to different settings. However, this study is a randomized controlled trial, which is considered the gold standard in the hierarchy of research designs for evaluating the efficacy and safety of a treatment intervention and each participant acts as their own control with two points of measurements. Moreover, the fact that each dietary pattern was personally designed for each patient taking into account sex, height, initial body weight and physical activity should also be highlighted. Finally, it is important point out that well-recognized healthy dietary patterns (AHA and RESMENA) should be considered of reasonable importance for precision nutrition as well as a role for LPC to understand lipid metabolism following weight loss in obese subjects with different degrees of liver disease.

## Conclusion

Lipidomic profile revealed a generalized decrease in circulating lysophospholipids, particularly LPC species, under energy restriction. The decrease in LPC was associated with improved BAAT score, after adjustment for weight loss, suggesting a role for these species concerning the association between LPC and liver status in obese individuals.

## Additional file

Additional file 1: **Figure S1.** Flowchart of participants. AHA, American Heart Association; RESMENA, metabolic syndrome reduction in Navarra; FLI, Fatty Liver Index. (DOCX 30 kb)

## Abbreviations

1H-MRS: 1H- Magnetic Resonance Spectroscopy
AHA: American Heart Associations
ALT: Alanine aminotransferase
AST: Aspartate aminotransferase
BAAT score: BMI, ALT, AGE and TG
BARD score: BMI, ALT/AST ratio, Diabetes
BMI: Body mass index
CHO: Carbohydrate
CV: Coefficient variation
Cytokeratin 18: M30 and M65 fragments
FA: Fatty acid
FLI: Fatty Liver Index
GGT: Gamma- glutamyl transferase
HCC: Hepatocellular carcinoma-
HDL-c: High density lipoprotein
HOMA-IR: Homeostasis model of Insulin resistance
HPLC: High Performance liquid chromatography
hsCRP: High sensitive Creative Reactive Protein
HSI: Hepatic steatosis index
IDF: International Diabetes Federation Criteria
LDL-c: Low density lipoprotein
LP: Lysophospholipids
LPC: Lysophosphatidylcholines
LPC-s: Lysophosphatidylcholines score
LPE: Lysophosphatidylethanolamine
LPE-s: Lysophosphatidylethanolamine score
LPI: Lysophosphatidylinositols
LPI-s: Lysophosphatidylinositols score
LPtotal: Lysophospholipids total score
MetS: Metabolic syndrome
MS: Mass spectrometry
NAFLD: Non-alcoholic fatty liver disease
NAFLD_LFS: Non-alcoholic fatty liver disease liver fat score
Q-TOF: Acurate Mass quadrupole Time of Flight
RESMENA: Reduction of Metabolic Syndrome in Navarra
TC: Total cholesterol
TCV: Total caloric value
TG: Triglycerides
TyG index: Triglycerides/glucose index
VAI: Visceral adipose index
VAT: Visceral adipose tissue-
WC: Waist circumference
