# Supplementary material for: Changes in lysophospholipids and liver status after weight loss: the RESMENA study
Source: Nutr Metab (Lond). 2018 Jul 17;15:51. doi: 10.1186/s12986-018-0288-5 (PMC6050739; doi:10.1186/s12986-018-0288-5)
Supplement: Supplementary file 1 — Figure S1. Flowchart of participants. AHA, American Heart Association; RESMENA, metabolic syndrome reduction in Navarra; FLI, Fatty Liver Index. (DOCX 30 kb) [file 12986_2018_288_MOESM1_ESM.docx]

Recruited simple

n= 109

AHA diet

Resmena group

n= 45

n= 48

12 excluded because did not present MetS according to the IDF criteria.

4 drop-out before starting

13 dropped out for missing data

13 dropped out for missing data

FLI calculation

n= 35

n= 32

n= 33

Baseline: High FLI 6-months: High FLI

n= 11

Baseline: High FLI 6-months: Low FLI

≥ 60 < 60

n= 12

Baseline: Low FLI 6-months: Low FLI

< 60 < 60

n= 10

≥ 60 ≥ 60

Figure 3. Flowchart of participants. AHA, American Heart Association; RESMENA, metabolic syndrome reduction in Navarra; FLI, Fatty Liver Index.
